# Fabrication and Wear Behavior of TiCN-Based Cermets with Nano-Diamond Addition

**DOI:** 10.3390/ma18235448

**Published:** 2025-12-03

**Authors:** Xiaoyong Ren, Xuyang Leng, Hong Deng, Guangxuan Yin

**Affiliations:** 1 School of Mechanical and Electrical Engineering, China University of Mining and Technology (Beijing), Beijing 100083, China; lxy6056@163.com (X.L.); denghong0716@163.com (H.D.); 15207125212@163.com (G.Y.); 2 Key Laboratory of Intelligent Mining and Robotics, Ministry of Emergency Management, Beijing 100083, China; 3 Zhengzhou Institute, China University of Geosciences (Beijing), Zhengzhou 451283, China

**Keywords:** TiCN-based cermets, nano-diamond, spark plasma sintering, mechanical properties, wear behavior

## Abstract

TiCN-based cermets have been widely used as cutting tools and wear-resistant coatings due to their excellent performance. New kinds of TiCN-based cermets that are being developed to have high performance have attracted extensive attention. In this work, TiCN-based cermets with nano-diamonds (NDs) as an additive were prepared by spark plasma sintering (SPS). The phase composition, microstructure, mechanical properties and wear behavior of the samples with different ND contents were systematically studied. The results show that a large fraction of the added nano-diamonds was transformed into graphite, while part of the diamond phase remained. The aggregation of the graphite became serious with more than 7 wt.% added nano-diamond. The relative density of the samples was approximately 87% and the hardness decreased with an increase in the added amount of nano-diamond. The average coefficient of friction of the samples ranged from 0.4 to 0.5. The graphite generated from nano-diamond lead to a deterioration in the mechanical properties of the prepared cermets and a reduction in their wear resistance. How to reduce the graphitization of diamond during the preparation of cermets should be considered in the follow-up study.

## 1. Introduction

TiCN-based cermets have become one of the preferred materials for preparing high-speed cutting tools because of their excellent hardness, even at high temperatures; outstanding wear resistance; and perfect chemical stability [1,2,3]. With the development of high-speed cutting technology, higher requirements are put forward for the performance of TiCN-based cermets. It has become one of the important goals of the researchers to further improve the mechanical properties and wear resistance of TiCN-based cermets [4,5].

Numerous attempts have been developed to enhance the mechanical properties of the cermets, such as using advanced sintering methods [6,7,8], optimizing the metal binder phase [6,9,10,11,12], and adding a hard reinforcement phase [13,14,15,16,17,18,19]. Spark plasma sintering (SPS) is one kind of advanced sintering technique that is commonly used in the preparation of cermets. During the sintering, plasma is generated on the powder surface, under the action of the DC pulse current, to achieve Joule heating [20,21]. At the same time, an axial pressure is applied to the sample. Therefore, SPS is often employed to fabricate high-performance cermets.

In order to further improve the wear resistance of cermets, composite strengthening is one of the main ways to improve the properties of TiCN-based cermets. Usually, the reinforcement phase uses ceramics with high hardness, such as TiN [13], ZrC [14], WC [15,18], Cr_3_C_2_ [16], Mo_2_C [17], TaC [17], NbC [19], TiB_2_ [21], AlN [22], and so on [23]. E. Shankar et al. [21] found that the TiCN-based cermets with a 15 wt.% added TiB_2_ showed a balanced enhancement in both fracture toughness and Vickers hardness. Diamond is currently the hardest known material. As a second-phase additive, it can effectively enhance the hardness and wear resistance of the material [24,25]. Then, due to the high sintering temperature of TiCN-based cermets and the presence of metal bonding phases such as Co or Ni, diamond is prone to graphitization during the sintering process [26]. The strengthening effect of carbon nanotubes or graphene on cermets have also been studied [27,28,29,30,31]. However, there are relatively few studies on diamond strengthening TiCN-based cermets. The unique Joule heating and axial pressure of SPS technology can effectively reduce the sintering temperature and time, which would reduce the graphitization of diamond.

Nano-diamond remains a potential reinforcement because of its extremely high hardness and modulus, although its graphitization in contact with Ni is thermodynamically favored. The rapid heating and short dwell time of SPS can reduce the driving force for this transformation, offering the possibility of retaining part of the diamond structure. Strategies such as lowering the peak temperature, using vacuum or inert atmospheres, or modifying the binder composition can further suppress graphitization. However, the behavior of nano-diamond in TiCN–Ni systems under SPS conditions has rarely been studied, which motivates the present work.

In this experiment, TiCN-based cermets with different ND contents were prepared by the spark plasma sintering process, with ultrafine TiCN powder as the raw material, WC as the sintering additive, and Ni as the metal binder, and the effects of the ND addition on the microstructure and mechanical properties of the TiCN-based cermets were studied. The friction coefficient, wear resistance, and wear mechanism of the TiCN-based cermets reinforced by diamond were studied.

## 2. Materials and Methods

### 2.1. Preparation

**Figure 1:**
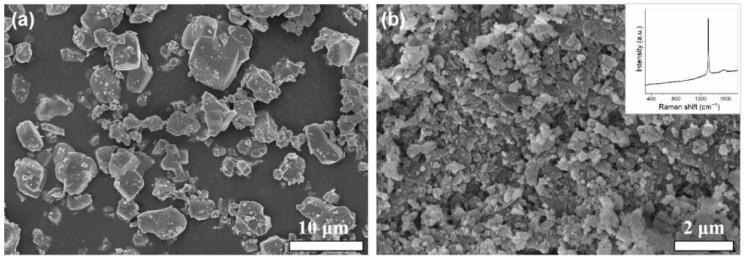
Typical SEM images of the starting powders: (**a**) TiCN and (**b**) nano-diamond. The inset in (**b**) shows the Raman spectrum of nano-diamond.

Commercially, TiCN, WC, and Ni powders are used as the starting powders to prepare TiCN-based cermets, and nano-diamond (ND) was used as the strengthening phase. Figure 1 shows the typical SEM images of the TiCN powder and nano-diamond powder. It can be seen that the shape of the TiCN powder particles is multi-angled, with an average size of ~5 μm. The size of the nano-diamond particles is about ~700 nm. The Raman spectrum of nano-diamond shows a strong Raman peak at 1332 cm^−1^, indicating that the main component of the powder is diamond.

Table 1 shows the chemical compositions of the designed samples. During the experiment, the powder mixture was milled using ZrO_2_ balls in ethanol at 200 rpm for 2 h, with a ball-to-powder ratio of 10:1, a total powder mass of 200 g, and a ball diameter of 5 mm, and ethanol medium in a planetary ball mill (XQM-0.4A, Tencan Powder, Changsha, China). The milled powder is dried in a blast-drying oven and then sieved. In the sintering process, the powder is placed in a graphite mold with an inner diameter of 15 mm and sintered in a spark plasma sintering furnace (SPS, LaBox-225, Sinter Land, Japan). After sintering, the samples are polished on the surface and then subjected to subsequent characterization tests.

The SPS was performed under a vacuum atmosphere (≈10 Pa). The powders were heated from room temperature to 1350 °C at a heating rate of 100 °C/min, followed by a dwell time of 5 min at the peak temperature. A pulsed DC current mode (on/off ratio 12:2) was applied, and the axial pressure of 50 MPa was maintained throughout the heating and holding stages. Prior to tribological testing, the surface roughness of the samples was controlled at Ra ≈ 0.05–0.07 μm to ensure consistent contact conditions.

### 2.2. Characterization

The actual density is measured by the Archimedes drainage method. Relative density is defined as the ratio of the actual density to the theoretical density. The theoretical density is calculated based on the principle of the powder mixture. An X-ray diffractometer (XRD, D8-Advance, Bruker, Bremen, Germany) is used to analyze the phase composition of the samples by using the continuous scanning mode within the 2θ range of 20–90° and a scanning speed of 5 °/min. The Vickers hardness tester (EV500-2A, Everone, Shanghai, China) is used to measure the hardness of samples with a load of 10 kgf and a holding time of 10 s. The hardness test is repeated at least five times for each sample to calculate the average value. The morphology is observed by a SU8220 cold field emission scanning electron microscope (SEM), with the energy-dispersive X-ray spectrometer (EDS). The Raman result is detected by a high-resolution Raman spectrometer (HR Evolution, Horiba, Longjumeau, France) with a laser wavelength of 532 nm. A universal micro-tribotester (UMT-3, Bruker, Germany) is used for conducting friction and wear tests in the reciprocating mode in a ball-on-disk contact configuration, under a load of 3 N, a frequency of 5 Hz, and a stroke of 4 mm. Si_3_N_4_ balls (4 mm in diameter and roughness, *R_a_*, of about 10 nm) is used as the friction pair. The friction test for each sample is repeated at least 3 times to calculate the average value. The friction coefficient curve was automatically recorded by the system. The wear traces on the samples were measured using a 3D white-light interferometer (Nexview, ZYGO Lamda, Middlefield, CT, USA).

## 3. Results and Discussion

### 3.1. Phase Composition and Microstructure

**Figure 2:**
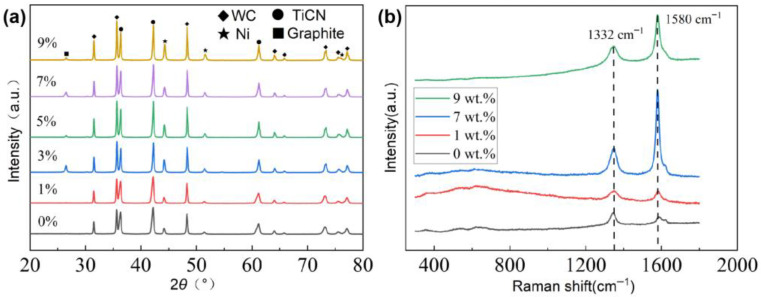
(**a**) XRD patterns and (**b**) Raman spectra of the prepared TiCN-based cermet with different contents of nano-diamond.

Figure 2 shows the XRD patterns and Raman spectra of the prepared TiCN-based cermets with different contents of nano-diamond. In the sample without the nano-diamond addition, the peaks of TiCN, WC, and Ni were detected. In the samples with the nano-diamond addition, the peaks of the graphite were detected in addition to the peaks of TiCN, WC, and Ni. As the amount of nano-diamond added increased, the intensity of the graphite peak gradually increased. The peaks of the diamond are not obvious in all the samples. The main reasons for the failure to detect the peaks of the diamond might be the low content of nano-diamond, the weaker peak intensity of diamond compared to that of TiCN and WC, and the graphitization of diamond during sintering. However, graphite peaks at 26.5° were detected in the samples with the diamond addition exceeding 3 wt.%, indicating that the nano-diamond underwent graphitization during the sintering process of the samples.

In the Raman spectra of samples with the nano-diamond addition, two main peaks were detected at the positions of 1332 cm^−1^ and 1580 cm^−1^. The peak at 1332 cm^−1^ is the characteristic peak of diamond and the peak at 1580 cm^−1^ is the characteristic peak of graphite. The Raman results indicated that a relatively severe graphitization of the nano-diamond occurred during the sintering process, which is consistent with the XRD results. A semi-quantitative analysis of the Raman spectra shows that the ID/IG ratios are 1.75, 1.22, 0.38, and 0.48 for 0, 1, 7, and 9 wt.% nano-diamond, respectively, demonstrating the strongest graphitization at 7 wt.% nano-diamond. The diamond peak was also detected in the Raman results, indicating that some of the diamond was retained in the prepared samples. The graphitization of diamond would increase the graphite phase in the sample. Due to the very low hardness and strength of the graphite, the graphite phase in the sample is like a pore defect, which will significantly reduce the hardness of the cermets. On the other hand, due to the excellent lubricating properties of graphite, the presence of the graphite phase may reduce the coefficient of friction of the cermets.

**Figure 3:**
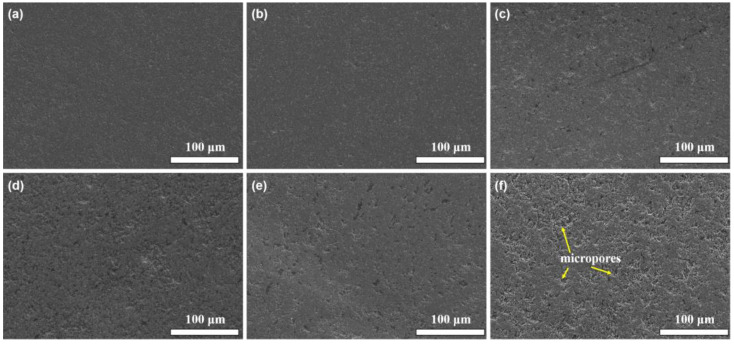
SEM images of the prepared TiCN-based cermets on the polished surfaces with different contents of added nano-diamond: (**a**) 0, (**b**) 1 wt.%, (**c**) 3 wt.%, (**d**) 5 wt.%, (**e**) 7 wt.%, and (**f**) 9 wt.%.

Figure 3 shows the typical SEM images of the prepared TiCN-based cermets on the polished surfaces, with different contents of added nano-diamond. It can be observed that with the increase in added nano-diamond, the polished surface of the samples becomes less smooth and many small pits appear. As the amount of nano-diamond added increases, more and more small pits appear. The pore-area quantification, based on the SEM images, shows that the porosity is approximately 0.45%, 0.82%, 1.74%, 3.86%, 7.92%, and 12.38% for the samples containing 0, 1, 3, 5, 7, and 9 wt.% nano-diamond, respectively. The generated graphite is prone to being ground off during the polishing process, resulting in the formation of pits.

**Figure 4:**
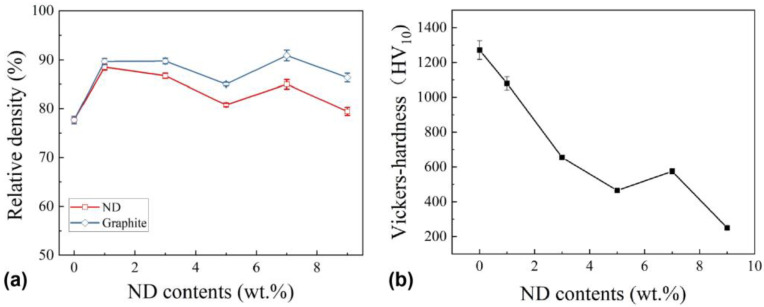
Relative density (**a**) and Vickers hardness (**b**) of the prepared TiCN-based cermets.

Figure 4a shows the calculated relative densities of the samples with different amounts of added nano-diamond. Relative density is defined as the ratio of the actual density to the theoretical density. Here, the theoretical density of the prepared sample is calculated based on the mixing principle. Through the previous characterization analysis, some of the nano-diamond transformed into graphite during the sintering process, and the proportion of the transformation is difficult to determine. Therefore, the theoretical density and the corresponding relative density of the samples were calculated based on the two extreme cases: the diamond completely transforming into graphite (graphite line in Figure 4a) and no transformation at all (ND line Figure 4a). The actual relative density was between the two curves. It can be seen from the figure that when the content of nano-diamond was 1–3 wt.%, the relative density of the samples slightly increased, up to about 87%, indicating that the sample prepared at this time was relatively dense. However, when the content of nano-diamond reached 5 wt.%, the relative density of the samples decreased. When the content of nano-diamond was 9 wt.%, the relative density of the material decreased sharply. For the sample with 1–3 wt.% added nano-diamond, this may play a role in supplementing the carbon source through graphitization and may have helped reduce decarburization, thereby potentially contributing to densification during sintering. However, when the diamond content continued to increase, too much graphite was generated, resulting in a decrease in the density of the sample.

Figure 4b shows the average Vickers hardness of TiCN-based cermets with different nano-diamond additions. It can be seen that as the added amount of nano-diamond increased, the Vickers hardness of the samples gradually decreased, reaching the minimum value of approximately 230 HV_10_ with 9 wt.% added nano-diamond. This hardness value was confirmed through repeated measurements at multiple surface locations, and the results exhibited only a narrow variation range. This is mainly because nano-diamond transforms into graphite during the sintering process, resulting in numerous defects in the sample and a decrease in its hardness.

### 3.2. Tribological Properties

**Figure 5:**
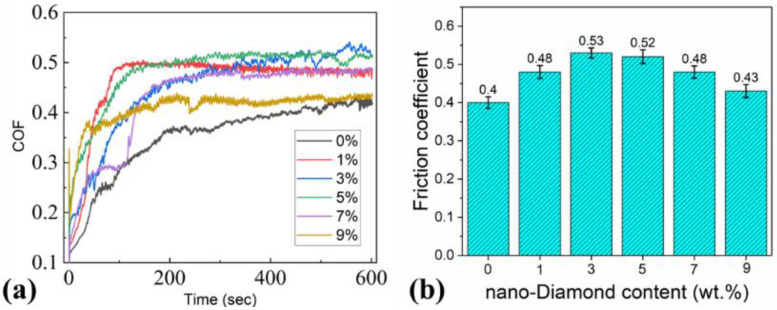
Variation in the (**a**) COF with time and (**b**) average friction coefficient with different nano-diamond contents of the prepared cermets.

The friction and wear tests were carried out by a universal micro-tribotester in the reciprocating mode in a ball-on-disk contact configuration, under a load of 3 N, a frequency of 5 Hz, and a stroke of 4 mm. The test was performed for 10 min, corresponding to 3000 reciprocating cycles and a total sliding distance of 24 m. The curve of the friction coefficient with time was used to illustrate the friction and wear performance (Figure 5a). Figure 5b shows the average friction coefficient with different nano-diamond contents of the prepared cermets. According to the experimental results, the friction coefficient of the sample without nano-diamond is the smallest, at about 0.4. The sample with 3 wt.% nano-diamond added shows the maximum coefficient of friction, at about 0.53. With the increase in the added amount of nano-diamond, the average coefficient of friction of the samples first increases and then decreases. When the added amount of nano-diamond is 9 wt.%, the coefficient of the friction is about 0.43.

**Figure 6:**
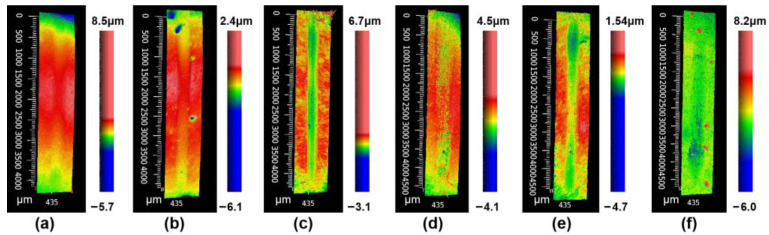
Wear morphology of the prepared (60 − x) TiCN-20WC-20Ni-xND cermets: (**a**) x = 0, (**b**) x = 1, (**c**) x = 3, (**d**) x = 5, (**e**) x = 7, and (**f**) x = 9.

**Figure 7:**
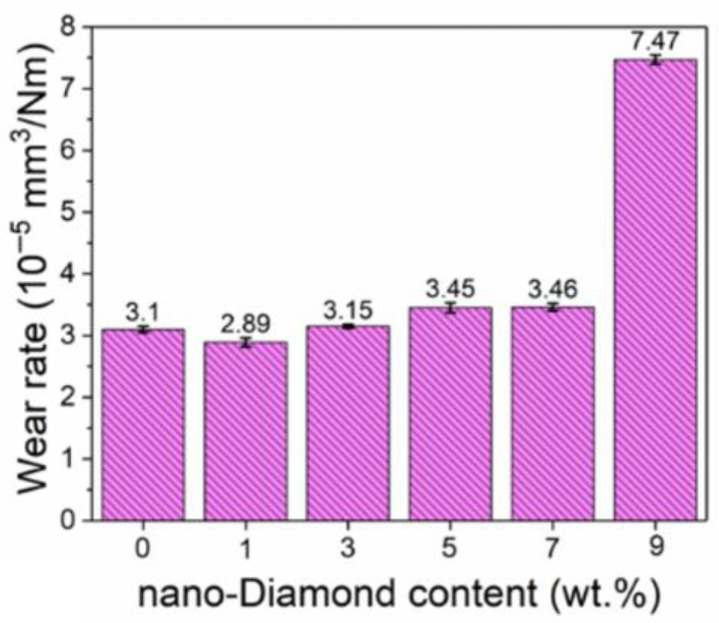
Average wear rate with different nano-diamond contents of the prepared cermets.

Figure 6 shows the wear morphology of the prepared samples by a 3D white-light interferometer. The long strip in the middle of the figure is the wear mark measurement area. Based on the wear morphology images, the wear volumes of the samples with different amounts of added nano-diamond were measured. Based on the measured wear volumes, the wear rate of the samples was calculated. Here, the wear rate is defined as the wear volume of per unit load and per unit wear distance. The wear rate of the samples is shown in Figure 7. The samples with a nano-diamond addition that did not exceed 7 wt.% exhibited similar wear rates., about 3.1 × 10^−5^ mm^3^/Nm. When the added content of nano-diamond reached 9 wt.%, the wear rate of the sample increased sharply to 7.47 × 10^−5^ mm^3^/Nm, which was about 2.4 times that of the sample with a smaller added amount of diamond. This increase is consistent with the progressive graphitization-induced hardness reduction observed at higher nano-diamond contents, which leads to a higher wear rate. This is mainly because the nano-diamond undergoes a relatively severe graphitization phenomenon during the sintering, which has been confirmed by both the results of XRD and Raman. Although the relative density of the samples did not change significantly with the increase in the added amount of nano-diamond, their hardness was greatly reduced because of the graphite generated from the diamond. The hardness of the sample with 9 wt.% nano-diamond was only 230 HV_10_. The low hardness of the sample led to a significant reduction in its wear resistance, resulting in a high wear rate. Although the graphite generated from diamond helps to reduce the friction coefficient of the sample to a certain extent, it is not sufficient to play a role in reducing the friction. Therefore, it is very important to prevent the graphitization of diamond during sintering.

**Figure 8:**
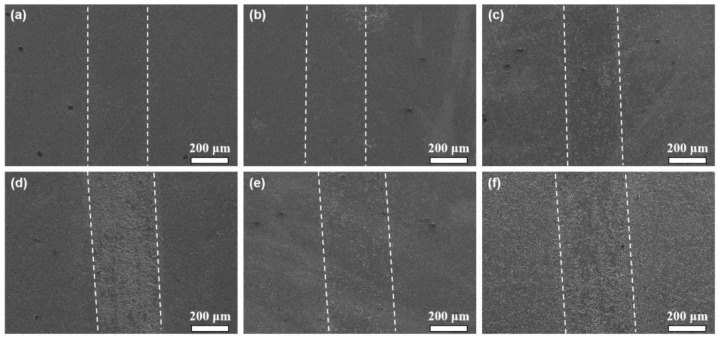
Wear mark morphology of the prepared (60 − x) TiCN-20WC-20Ni-xND cermets: (**a**) x = 0, (**b**) x = 1, (**c**) x = 3, (**d**) x = 5, (**e**) x = 7, and (**f**) x = 9.

Figure 8 shows the wear mark morphology of the prepared samples. The width of the wear marks ranged from 220 to 300 μm. When the nano-diamond addition content is less than 3 wt.%, the wear marks of the sample are relatively smooth. However, with the further increase in the content of nano-diamond, a lot of the pits appeared on the wear surface. The relatively smooth surface of the grinding marks is mainly attributed to the high density and hardness of the sample. However, when a large amount of diamond is added, a considerable amount of graphite is generated inside the sample, causing a sharp drop in the hardness of the sample. During the friction and wear procedure, the areas with graphite on the surface are prone to peeling off and forming pits, which increase the wear rate of the sample.

**Figure 9:**
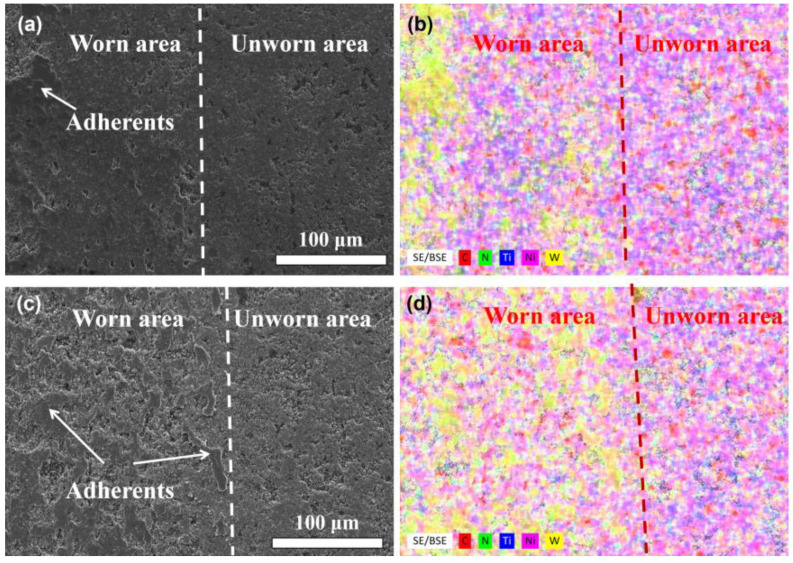
Distribution difference in elements inside and outside the wear marks of the prepared (60 − x) TiCN-20WC-20Ni-xND cermet: (**a**,**b**) x = 7, (**c**,**d**) x = 9.

On the other hand, some adherents can be seen on the wear marks of the samples with more than 3 wt.% added nano-diamond. These adherents are more like the formation of abrasive debris being crushed and deformed on the worn surface. Figure 9 shows the magnified images of the worn surfaces and the corresponding EDS mapping spectra of the samples with 7 and 9 wt.% added nano-diamond. The peeling pits and adherents can be seen more clearly by comparing the worn areas with the unworn areas. The morphology of the adherents also proves that they are formed due to the crushing and plastic deformation of grinding the debris.

By comparing the EDS mapping spectra with the corresponding SEM images, it can be seen that the main element of the peeling pits is carbon (C), indicating that the peeling pits area is mainly a rich zone of graphite. Due to the low hardness of graphite, it peels off during the wear procedure, forming the peeling pits. The pits accelerated the wear of the samples in this experiment. However, the pits may store lubricating fluid to promote lubrication when the sample operates under the fluid lubrication conditions. In the adherent region, the main enriched element is tungsten (W), as shown in Figure 9b,d. The envisioned graphite lubricating layer was not clearly detected on the surface of the sample, indicating that the graphite formed by adding nano-diamond did not play a good lubricating role during the lubrication. According to previous studies, the formation of a continuous graphite-rich tribofilm is generally expected to reduce the coefficient of friction due to its low shear strength. However, in the present work, no such coherent lubricating film was observed. Instead, a closer examination of the worn surfaces reveals that the graphite phase is distributed discontinuously in the form of isolated C-rich pits and patches, which prevents the development of a stable tribochemical layer. Such discontinuity eliminates the friction-reducing capability that is normally associated with graphite. The identification of these peeling pits as graphite-enriched regions is based on their relative C enhancement compared with the surrounding TiCN matrix, accompanied by correspondingly reduced Ti and N signals, indicating local depletion of the TiCN phase and the accumulation of graphite generated during the nano-diamond transformation.

Moreover, the EDS analysis indicates migration, detachment, and fragmentation of WC particles, which are released from the weakened TiCN–graphite interfaces and behave as hard abrasive debris. These combined effects—interfacial decohesion, graphite-induced pit formation, and the presence of WC-derived abrasive particles—demonstrate that, under the present sintering and sliding conditions, graphite acts more as a defect- or pit-generating phase, rather than an effective solid lubricant. This behavior explains the increase in COF and the aggravated wear damage observed at higher nano-diamond contents, despite the intrinsic lubricity of graphite. This was consistent with the result of the friction coefficient in Figure 5. The main source of the W on the worn surface was the WC added to the sample. The WC has high hardness and good wear resistance, but in the presence of graphite, its bonding with the matrix is poor. During the wear process, the WC is prone to forming hard grinding debris. In the subsequent wear process, due to the high temperature generated by friction, the WC surface will undergo partial oxidation and adhere to the wear surface, forming a tungsten-rich adherent.

## 4. Conclusions

TiCN-based cermets with nano-diamond as an additive have been prepared via the SPS method and their microstructure, phase composition, and tribological properties were studied. The following conclusions were obtained.

(1) During the sintering process, a large fraction of the added nano-diamonds was transformed into graphite. With the increase in the added amount of nano-diamond, the aggregation of the graphite became serious.
(2) The relative densities of the added nano-diamond samples were approximately 87%, and the compactness needs to be further improved. The hardness decreases with the increase in the added amount of nano-diamond.
(3) Using Si_3_N_4_ as the friction pair, the average coefficient of the friction of the samples ranged from 0.4 to 0.5. The coefficient of friction did not show a significant decrease after adding the nano-diamond. The wear rate of the samples with less than 7 wt.% of added nano-diamond was similar to that of the samples without added nano-diamond. However, when the added amount of nano-diamond was further increased, the wear rate of the samples increased sharply. The graphitization of nano-diamond during sintering is the main cause of the deterioration of the mechanical properties of composite materials.

In addition to the above conclusions, several practical strategies may be considered in future studies to mitigate the graphitization of nano-diamond during SPS. These include lowering the sintering temperature or reducing the dwell time to limit carbon diffusion, employing inert or vacuum atmospheres to suppress carbon activity, replacing Ni with binders that have a lower carbon affinity such as Co or FeAl, and incorporating carbon-scavenging additives such as B_4_C. Implementing such approaches may help maintain the stability of nano-diamond and improve the mechanical and tribological performance of TiCN-based cermets.

## Figures and Tables

**Table 1:** Chemical composition design (wt.%) of the developed cermets.

Cermets	TiCN	WC	Ni	Nano-Diamond
0%	60	20	20	-
1%	59	20	20	1
3%	57	20	20	3
5%	55	20	20	5
7%	53	20	20	7
9%	51	20	20	9

## Data Availability

The original contributions presented in this study are included in the article. Further inquiries can be directed to the corresponding author.
